# Recalibrate concepts of epigenetic aging clocks in human health

**DOI:** 10.18632/aging.206027

**Published:** 2024-07-17

**Authors:** Ze Zhang, Brock C. Christensen, Lucas A. Salas

**Affiliations:** 1 Divisons of Population Sciences and Medical Oncology, Dana-Farber Cancer Institute, Boston, MA 02115, USA; 2 Harvard Medical School, Boston, MA 02115, USA; 3 Department of Epidemiology, Geisel School of Medicine, Dartmouth College, Lebanon, NH 03755, USA; 4 Dartmouth Cancer Center, Dartmouth-Hitchcock Medical Center, Lebanon, NH 03766, USA; 5 Department of Molecular and Systems Biology, Geisel School of Medicine, Dartmouth College, Lebanon, NH 03755, USA

**Keywords:** epigenetics, DNA methylation, epigenetic clock, epigenetic age acceleration, methylation cytometry

**Figure 1:**
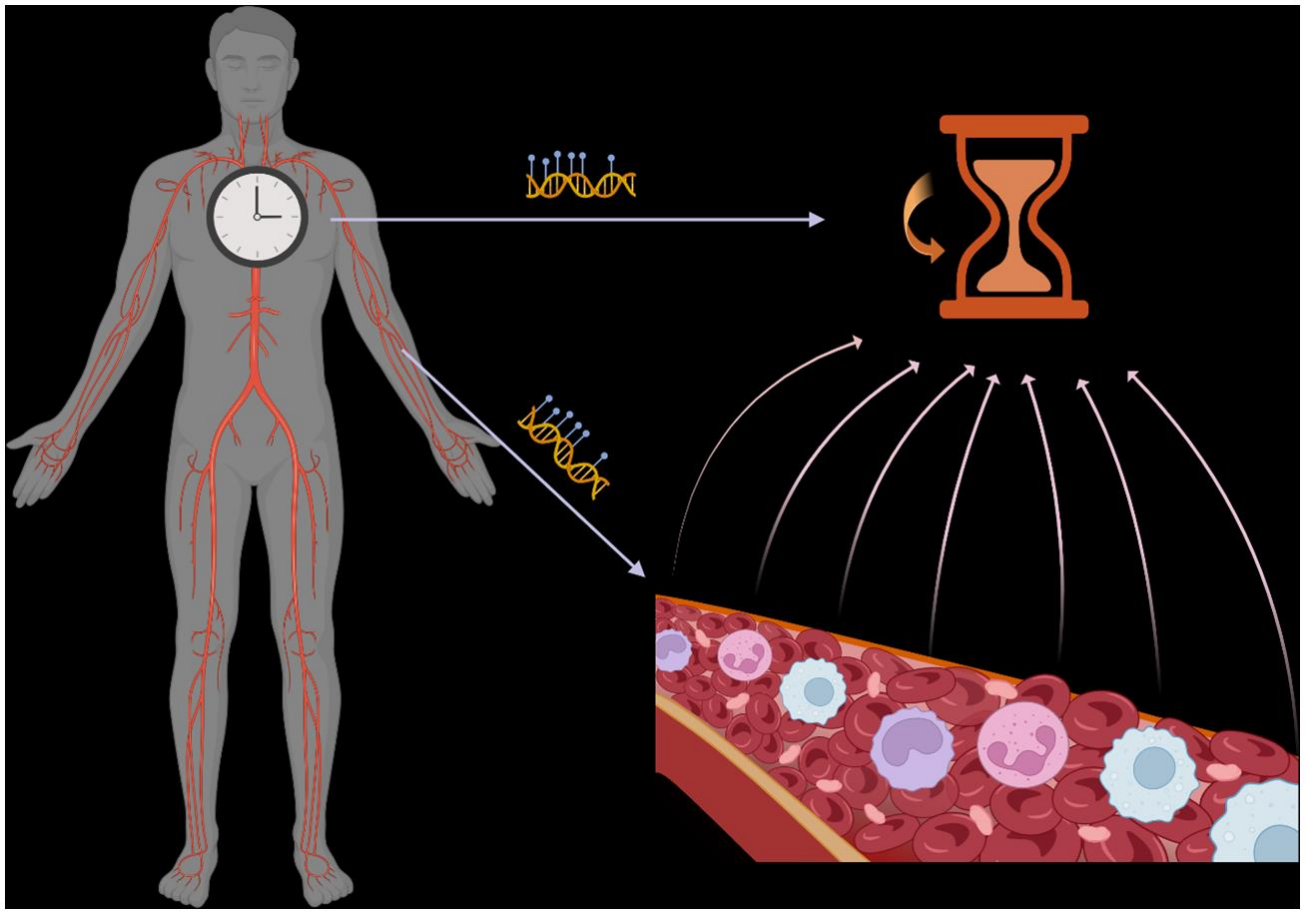
**Epigenetic clocks reflect the average DNA methylation signal across multiple cell types/states in a tissue.** Variations in this composition impact the interpretation of such clocks.

DNA methylation-based epigenetic clocks are used as biomarkers of biological age in human health. Multiple epigenetic clocks have rapidly emerged in the past decade by modeling DNA methylation changes with age in large cohorts, primarily using peripheral blood samples [1]. The difference between chronological and biological age, termed biological or epigenetic age acceleration (EAA), represents a valuable biomarker for disease risk assessment. Studies have shown strong associations between EAA and various health outcomes, including disease risk and mortality [2]. Despite efforts to understand the functional implications of features used to estimate biological age, the underlying mechanisms of these clocks remain poorly understood, leading to potential misinterpretations of their associations with health outcomes. While clocks exist for multiple tissues, blood-based clocks are more numerous and widely applied because blood biospecimens are commonly used to measure DNA methylation data in human studies, including large cohorts. Epigenetic clocks can be dissected into intrinsic and extrinsic age effects on DNA methylation. Although some studies incorporate partial cell type composition information when analyzing EAA, previous deconvolution technologies have limited the number of cell types included. Leveraging high-resolution methylation cytometry in blood samples [3], we recently studied the association of 12 immune cell types with EAA in healthy and diseased populations, shedding light on the complex interplay between immune cell composition and epigenetic aging [4] (Figure 1).

Our study revealed that the composition of immune cells significantly affects the variation in EAA across six common blood epigenetic clocks. Differences in naïve and memory lymphocyte compartments are key drivers of this variation. We then employed a new method to calculate intrinsic epigenetic age acceleration (IEAA) in blood, adjusting immune cell composition to an unprecedentedly detailed level, including multiple naïve and memory lymphocyte compartments. Applying this method to a rheumatoid arthritis cohort, we demonstrated the impact of adjusting for cell types on EAA differences. We recommend calculating IEAA with major naïve and memory cells adjusted to minimize confounding effects and directly examining immune cell composition within specific conditions for a more comprehensive understanding of their impact on the immune system.

In this editorial, we aim to address the key implications of our study on epigenetic aging clocks in human health from a broader perspective. While epigenetic clocks are widely hyped as aging biomarkers today, it's essential to recalibrate some fundamental concepts in this field.

First, epigenetic aging in human health should be viewed holistically. The relationship between epigenetic age and health outcomes is often viewed through a narrow lens. Blood epigenetic clocks are predominantly used due to their availability. However, as measured by these clocks, studies and trials frequently focus solely on blood, neglecting broader aging effects on the entire organism. Our study underscores the intricate link between immune cell composition and epigenetic age in blood. Nonetheless, limitations in technologies like epigenetic clocks and cell-type deconvolution methods hinder our understanding of epigenetic age in other tissues and organs, limiting the overall perspective. Furthermore, this issue can extend beyond scientific studies into commercial products. Products based on epigenetic clocks often claim to measure biological age using noninvasive samples such as saliva or buccal swabs. However, they frequently fail to acknowledge or interpret the broader biological implications of these results on health-related outcomes. This oversight can lead to exaggerated claims and misconceptions among the public. A younger biological age based on a buccal swab does not necessarily equate to a younger immune system or skeletal muscle composition.

Second, accept the limitations of epigenetic aging clocks. While efforts have been made to develop clocks independent of tissue or cell type, our study demonstrates that entirely eliminating the confounding effects of cell type in EAA calculation is nearly impossible. Senescence at the cellular level manifests differently across cell types and states, making it challenging to devise a single clock applicable to all cell types. Although universal epigenetic aging marks may exist, modeling them accurately across cell types at the bulk tissue level is complicated by age-related changes in cell composition. Ideally, biospecimen-specific and cell type-specific clocks should be developed to track aging at the cellular level. However, this approach requires additional resources, including time, money, and labor, detracting from the convenience of measuring biological age using bulk samples. Moreover, pan-tissue epigenetic clock development faces challenges due to inadequate training samples, with blood samples predominating in training datasets. This bias towards blood may limit the generalizability of pan-tissue clocks. Notably, a recent publication proposed measuring organ-specific aging using levels of human blood plasma proteins originating from specific organs. This innovative approach allows for tracing pan-tissue aging processes using noninvasive samples, offering a promising alternative to DNA methylation-based clocks [5].

Third, contextualize when interpreting epigenetic aging results. The perception that an accelerated epigenetic age is always detrimental stems from a limited understanding of the clock mechanism. Contrary to this notion, multiple studies have documented beneficial outcomes in specific groups of cancer patients with accelerated epigenetic age [6]. Immune responses play a pivotal role in cancer patient outcomes, and the connection between immune cell composition and epigenetic age established in our study sheds light on how epigenetic age acceleration affects these outcomes, especially in treatments like chemotherapy, radiation therapy, and immunotherapy that influence or rely on the immune system. Moreover, research has revealed significant daily oscillations in epigenetic age, mirroring changes in immune cell composition over the circadian rhythm [7]. Seasonal fluctuations in blood cells further suggest a potential seasonal effect on epigenetic age acceleration in blood [8]. Therefore, contextualization is essential to grasp the whole picture when interpreting epigenetic age acceleration in health-related outcomes.

Fourth, model epigenetic biomarkers on biological pathways to avoid black boxes. While direct modeling of DNA methylation changes with age enhances the predictability of biological age, it often obscures specific pathways captured by the algorithm, resulting in black boxes. To mitigate this, further efforts should focus on directly modeling aging and senescence-related pathways for novel biomarkers tracing age. Several markers exemplify the value of this approach. EpiTOC, for instance, functions as a mitotic clock, estimating stem cell divisions [9]. It tracks age and correlates with increased cancer risk, offering insights into the association between cellular aging processes and cancer. Additionally, the fetal cell origin (FCO) DNA methylation signature delineates the lineage of cells from fetal to adult stages, capturing age-related changes in early development and detecting abnormal development at an early stage [10]. Furthermore, deep-learning methodologies, *MethylCapsNet* and *MethylSPWNet*, allow the incorporation of prior biological knowledge to construct biologically informed predictors of outcomes like age [11]. These epigenetic clocks offer clear and straightforward biological implications for health-related outcomes, enabling a deeper understanding of aging processes and associated risks.

We hope our study and this editorial provide valuable insights into interpreting epigenetic aging clocks in future research and, most importantly, recalibrate essential concepts surrounding epigenetic aging clocks and their implications for human health.
